# Supplementary material for: IL-21 in Conjunction with Anti-CD40 and IL-4 Constitutes a Potent Polyclonal B Cell Stimulator for Monitoring Antigen-Specific Memory B Cells
Source: Cells. 2020 Feb 13;9(2):433. doi: 10.3390/cells9020433 (PMC7072853; doi:10.3390/cells9020433)
Supplement: Supplementary file 1 [file cells-09-00433-s001.pdf]

Supplementary Materials:

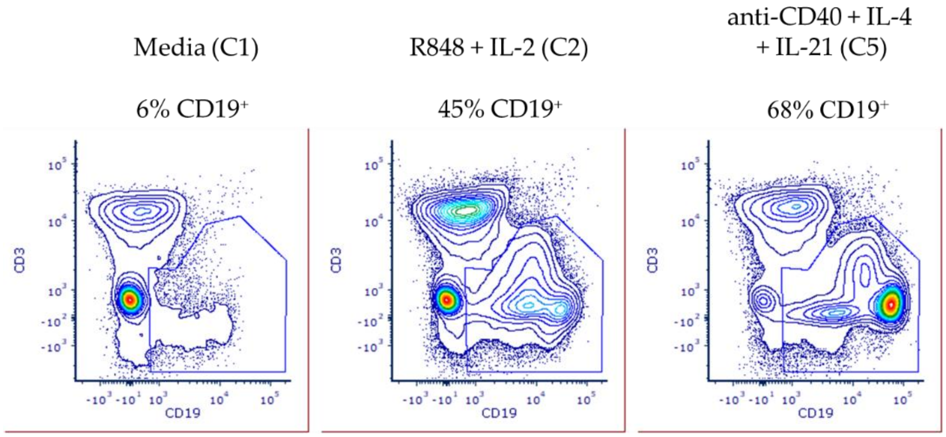

Supplementary Figure 1. Expansion of B cell frequency following *in vitro* stimulation. Representative flow cytometry plots depicting frequency of viable B cells ($CD19^+$) after 5 days of *in vitro* culture in media alone (C1), R848 + IL-2 (C2) or anti-CD40 + IL-4 + IL-21 (C5).

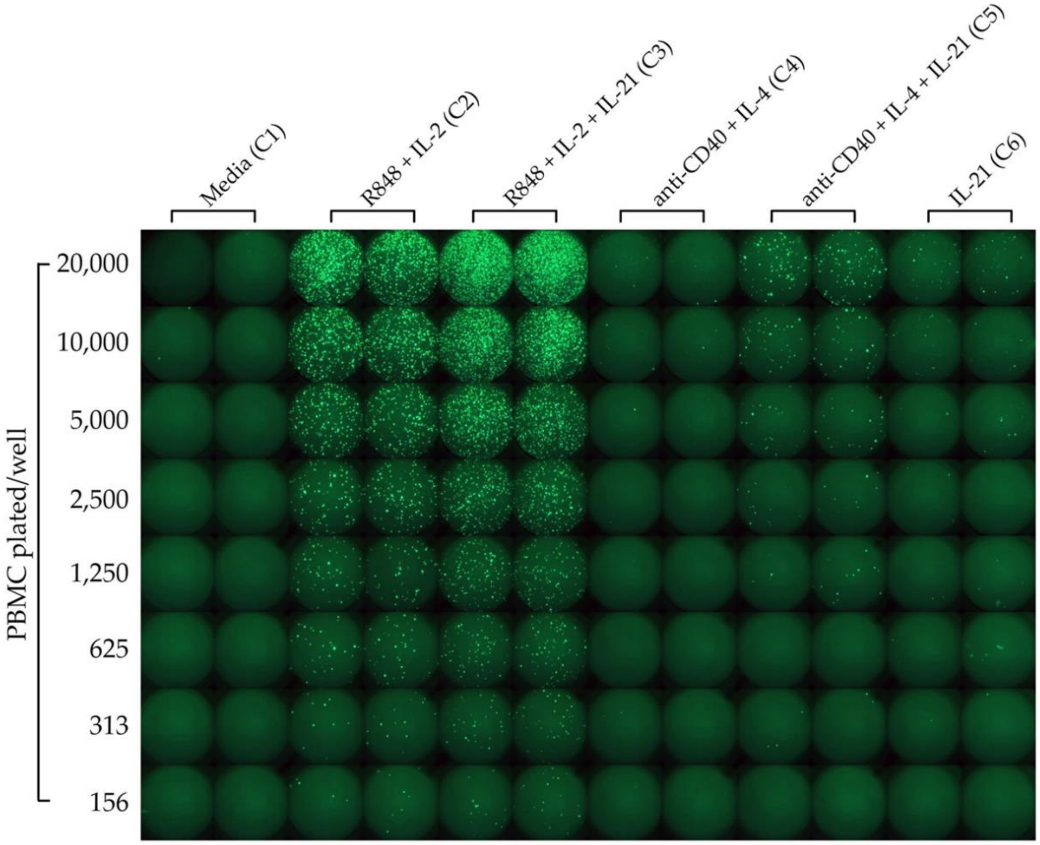

Supplementary Figure 2. Serial dilution of *in vitro* activated IgM+ ASC. Representative IgM+ ImmunoSpot® assay depicting results obtained following *in vitro* stimulation under each of the specified six culture conditions (detailed in *Materials and Methods*). Cell inputs are indicated next to the corresponding rows on the left side of the plate overview image. Each condition was tested in duplicate wells.

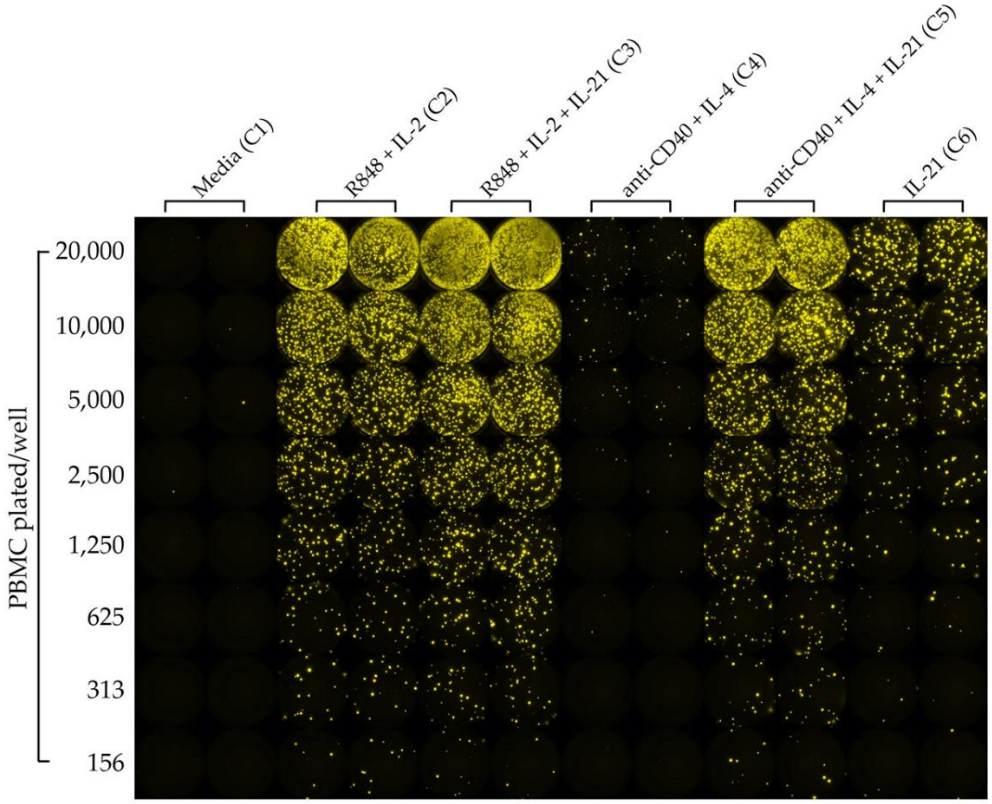

Supplementary Figure 3. Serial dilution of *in vitro* activated IgG+ ASC. Representative IgG+ ImmunoSpot® assay depicting results obtained following *in vitro* stimulation under each of the specified six culture conditions (detailed in *Materials and Methods).* Cell inputs are indicated next to the corresponding rows on the left side of the plate overview image. Each condition was tested in duplicate wells.

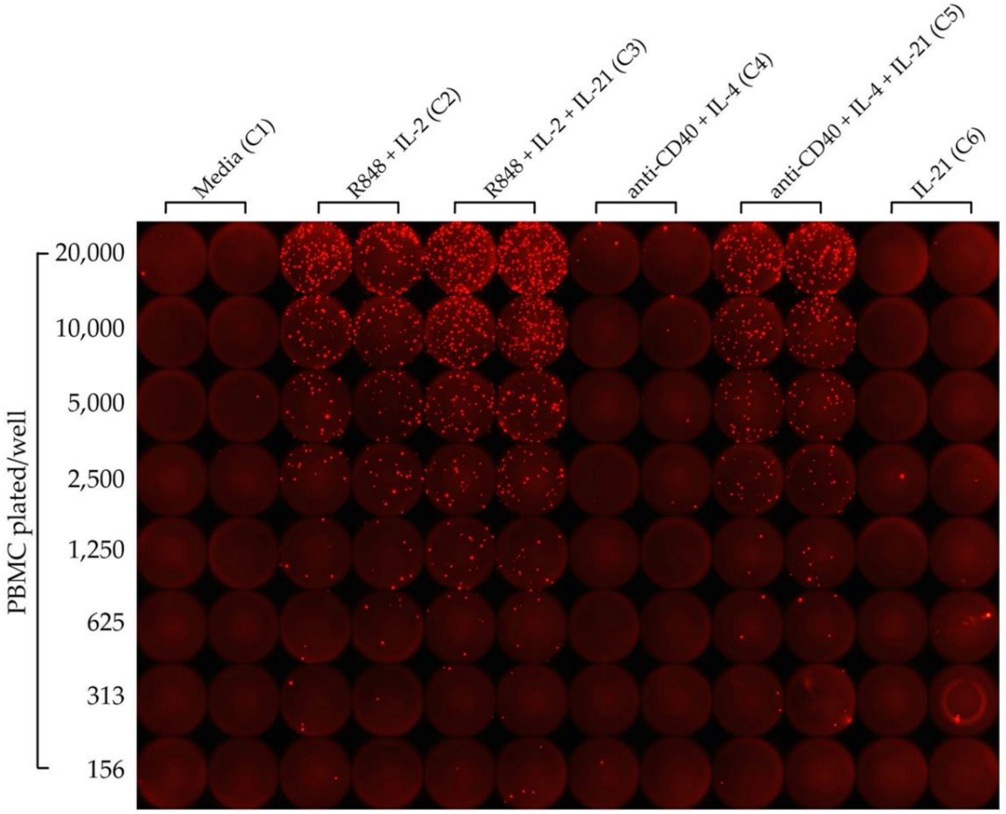

Supplementary Figure 4. Serial dilution of *in vitro* activated IgA⁺ ASC. Representative IgA⁺ ImmunoSpot® assay depicting results obtained following *in vitro* stimulation under each of the specified six culture conditions (detailed in *Materials and Methods).* Cell inputs are indicated next to the corresponding rows on the left side of the plate overview image. Each condition was tested in duplicate wells.

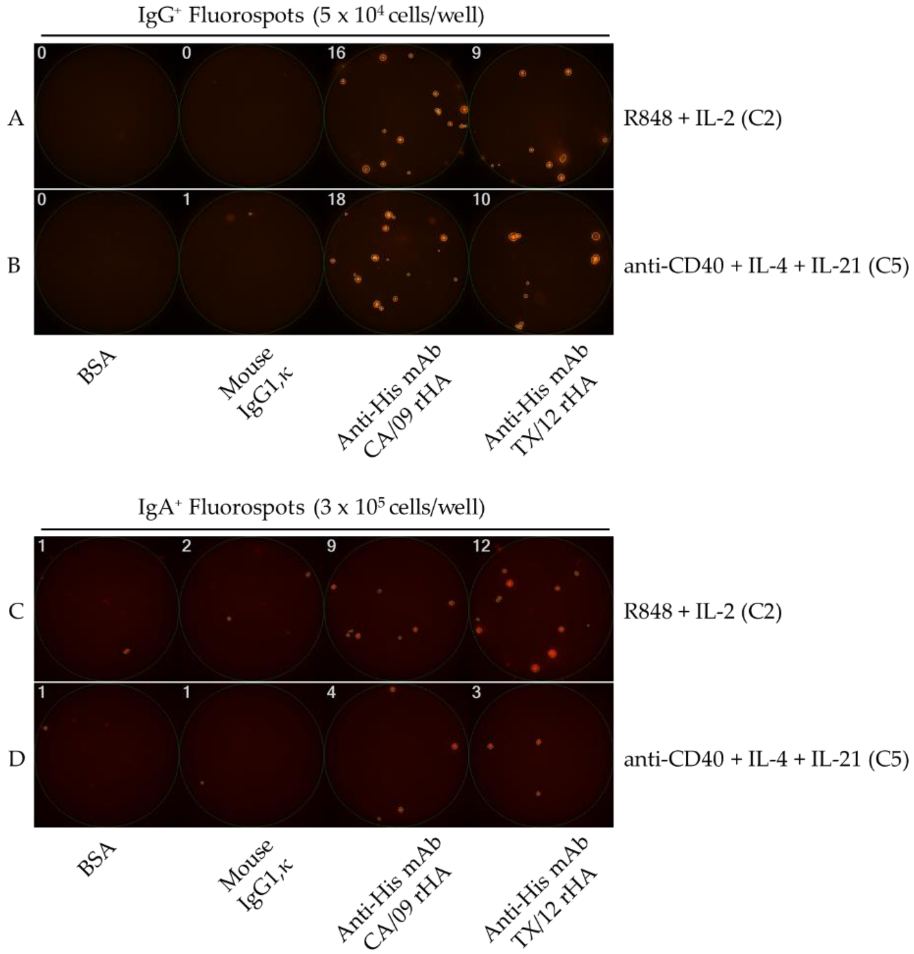

Supplementary Figure 5. Specificity controls for influenza-specific assays. Representative IgG^+ (panels A and B) or IgA^+ (panels C and D) ImmunoSpot® images obtained following *in vitro* stimulation of donor PBMC with R848 + IL-2 (C2) or anti-CD40 + IL-4 + IL-21 (C5). Negative control wells were coated with 10ug/mL bovine serum albumin (BSA) or irrelevant mouse IgG1, κ monoclonal antibody (mAb). Influenza-specific wells were first coated with 10ug/mL mouse anti-His mAb and then with recombinant HA proteins representing the H1N1 (A/California/07/2009, CA/09) or H3N2 (A/Texas/50/2012, TX/12) influenza vaccine strains (detailed in *Materials and Methods).*
